# Polymorphic *Alu* Insertion/Deletion in Different Caste and Tribal Populations from South India

**DOI:** 10.1371/journal.pone.0157468

**Published:** 2016-06-17

**Authors:** Rathika Chinniah, Murali Vijayan, Manikandan Thirunavukkarasu, Dhivakar Mani, Kamaraj Raju, Padma Malini Ravi, Ramgopal Sivanadham, Kandeepan C, Mahalakshmi N, Balakrishnan Karuppiah

**Affiliations:** 1 Department of Immunology, School of Biological Sciences, Madurai Kamaraj University, Madurai, 625 021, Tamil Nadu, India; 2 Department of Biotechnology & Genetic Engineering, School of Biotechnology, Bharathidasan University, Tiruchirappalli, 620 024, Tamil Nadu, India; 3 Department of Zoology, A.P.A.College of Arts & Culture, Palani, 624 601, Tamil Nadu, India; 4 Regional Forensic Science Laboratory, Madurai, 625 020, Tamil Nadu, India; Texas Tech University Health Science Centers, UNITED STATES

## Abstract

Seven human-specific *Alu* markers were studied in 574 unrelated individuals from 10 endogamous groups and 2 hill tribes of Tamil Nadu and Kerala states. DNA was isolated, amplified by PCR-SSP, and subjected to agarose gel electrophoresis, and genotypes were assigned for various *Alu* loci. Average heterozygosity among caste populations was in the range of 0.292–0.468. Among tribes, the average heterozygosity was higher for Paliyan (0.3759) than for Kani (0.2915). Frequency differences were prominent in all loci studied except *Alu* CD4. For *Alu* CD4, the frequency was 0.0363 in Yadavas, a traditional pastoral and herd maintaining population, and 0.2439 in Narikuravars, a nomadic gypsy population. The overall genetic difference (Gst) of 12 populations (castes and tribes) studied was 3.6%, which corresponds to the Gst values of 3.6% recorded earlier for Western Asian populations. Thus, our study confirms the genetic similarities between West Asian populations and South Indian castes and tribes and supported the large scale coastal migrations from Africa into India through West Asia. However, the average genetic difference (Gst) of Kani and Paliyan tribes with other South Indian tribes studied earlier was 8.3%. The average Gst of combined South and North Indian Tribes (CSNIT) was 9.5%. Neighbor joining tree constructed showed close proximity of Kani and Paliyan tribal groups to the other two South Indian tribes, Toda and Irula of Nilgiri hills studied earlier. Further, the analysis revealed the affinities among populations and confirmed the presence of North and South India specific lineages. Our findings have documented the highly diverse (micro differentiated) nature of South Indian tribes, predominantly due to isolation, than the endogamous population groups of South India. Thus, our study firmly established the genetic relationship of South Indian castes and tribes and supported the proposed large scale ancestral migrations from Africa, particularly into South India through West Asian corridor.

## Introduction

India is served as the important corridors for human migration and evolution. A small group of modern humans ventured ‘out-of-Africa’ through the southern coastal route to colonize the Middle East, India, Southeast Asia, Australia and subsequently the other parts of the globe [1,2]. The Indian populations are stratified as tribes and castes. In India approximately 4,635 populations, among which 532 are tribes, including 72 primitive tribes (including 36 hunter- gatherers) [3]. The tribal group constitutes about 8% of the total Indian population [4]. The Tamil Nadu population can be divided based on migrational history, genetics and anthropology [5]. According to 2011 census reported, of the 72.14 million and 7.2 lakhs of Tribal population. The majority of the population groups of Tamil Nadu belongs to Proto-Australoid ethnicity. The Indo-Aryan people of northern India were considered to be members of the White race; the southern Indian people were biologically distinct Indo-Dravidian race, also known variously known by anthropologists as Veddoid, Indigenous Australians or Palaeo-Indid. Thus, the people of India are a blend of Whites, Central and East Asians and Indigenous Australians (Aboriginal peoples) races. Reich et al. reported that an ‘ancestral North Indian (ANI)’ population shared 30–70% similarities of Middle East, Central Asia and Europe and an ‘ancestral South Indian (ASI)’, has no relation with any population outside of India [6].

The genomic variations among individuals may help to understand the evolutionary and migrational course of populations. The genetic variations and/or polymorphism at loci that code for expressed profess are commonly deleterious and therefore, are often negatively selected and hence eliminated. On the other hand, allelic polymorphisms, especially in the non-coding regions of the human genome are expected to be evolutionarily neutral. Of late, several insertion/deletion polymorphism have been discovered in the human genome. *Alu* sequences are thought to be ancestrally derived from 7SL RNA gene was mobilized through a RNA polymerase III derived transcript by a process called ‘retro position’ process [7, 8]. *Alu* insertion polymorphisms identify the patterns of human genetic diversity and history, race determination, gender identification, personal identification, paternity testing. *Alu* insertional elements are a family of SINEs and presence of an *Alu*I recognition site in the sequence [9]. The human genome contains 1,100,000 *Alu* repeats, which represents ~11% of nuclear DNA [10]. It is often located in non-coding regions (intergenic spacers and introns) [11, 12]. *Alu* insertions are 300 bp length, dimeric in structure, composed of 3’ oligo (dA)-rich tail and short flanking repeats [13–15]. The insertion *Alu* polymorphism has an important application in phylogenetic analyses of human populations [16–18, 19]. To determine the genetic differentiation among populations, Gst values (a measure of the interpopulation variability), Ht (a measure of genetic variability in total populations) and Hs (a measure of Intra population of genetic variability) for each polymorphic locus were determined. A number of ‘*Alu*’ polymorphic loci were previously been studied for many Indian populations [20, 21, 22, 23]. However, studies on South Indian castes and tribes are meager [24, 25, 26, 27, 28]. The present study is an attempt to analyze the seven polymorphic autosomal DNA loci such as *Alu* ACE, *Alu* TPA25, *Alu* FXIIIB, *Alu* Apo, *Alu* D1, *Alu* Pv92 and *Alu* CD4 among castes and tribes of the state of Tamil Nadu, South India.

## Materials and Methods

### Population Samples and Autosomal Markers

**Fig 1:**
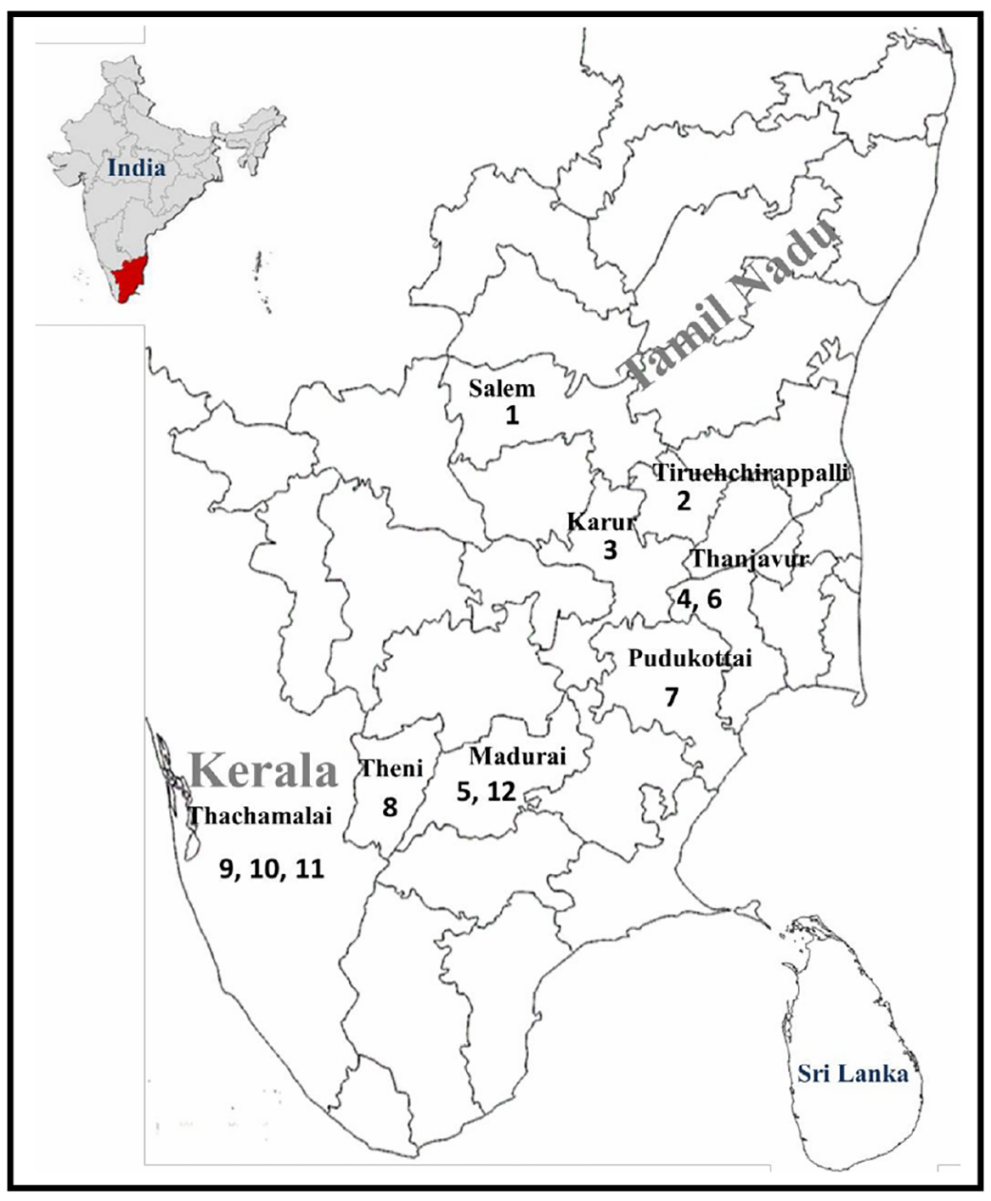
Map showing study areas of caste and tribal populations. (1) Vanniyar (n = 51); (2) Pallar (n = 50); (3) Vettuva Gounder (n = 44); (4) Kallar (n = 53); (5) Iyer (n = 44); (6) Sourashtra (n = 39); (7) Narikuravar (n = 58); (8) Paliyar (n = 58); (9) Namboothiri (n = 35); (10) Nair (n = 56); (11) Kani (n = 49); (12) Yadava (n = 54).

5 ml of blood samples were collected from 574 unrelated volunteers from twelve different population groups from South India. The populations selected for the present study includes Pallan, Nair (Kerala) Namboothiri (Kerala), Kani, Vanniyar, Paliyar, Narikuravar, Sourashtra, Iyer, Vettuva Gounder, Kallar and Yadava. They belong to different geographical locations in the states of Tamil Nadu and Kerala. The sample size, location of sampling and anthropological information was given in Fig 1. The Ethnographical notes of the studied population were listed in S1 File. Institutional ethical clearance was obtained from Madurai Kamaraj University Ethical and Review Board Committee (ERC) and the informed written consent was obtained from all the individuals who participated in the study which includes demographical details such as age, gender and family history for major illness or disease if any.

The DNA samples were extracted from peripheral blood lymphocytes using a standard salting-out procedure [29]. Each DNA samples were amplified by polymerase chain reaction (PCR) using locus-specific primers for the insertion-deletion polymorphism of seven *Alu* elements (*Alu* ACE, *Alu* TPA25, *Alu* FXIIIB, *Alu* CD4, *Alu* APO, *Alu* D1, *Alu* PV92). The protocols for these markers have been described elsewhere [20–22, 30] (S1 Table). Amplified PCR products were run on agarose gel and visualized under UV light.

### Statistical analysis

Allele frequencies were calculated by direct counting for each population. Heterozygosities at individual loci and the overall average heterozygosity were calculated by using the allele frequencies for each population. Hardy-Weinberg equilibrium was tested using a χ^2^ goodness of fit test, with Bonferroni’s correction for multiple comparisons. The dendrograms were constructed by neighbourjoining (NJ) method [31]. Principal Component Analysis (PCA) was performed to generate a distances between populations using the raw data of allele frequencies by means of the NTSYS (Numerical Taxonomy and Multivariate Analysis System).

## Results

### Allele Frequencies in Tribal Populations

The allele frequencies and heterozygosities for the insertion (+) and/or deletion (-) alleles for two tribal populations, Kani and Paliyar (Nilgiri hills, Western Ghats, Tamil Nadu) are presented in Table 1. Many of the loci studied revealed a higher level of heterozygosity in two tribal populations studied. Out of the 7 loci studied, heterozygosity was similar for loci ACE (0.5918 vs. 0.5614), FXIIIB (0.2244 vs. 0.2105) and CD4 (0.1224 vs. 0.1754) and highly varied for loci TPA25 (0.3265 vs. 0.5087), APO (0.1428 vs. 0.3508), D1 (0.1020 vs. 0.3859) and PV92 (0.5306 vs. 0.4385). Interestingly, the average heterozygosity is higher in Paliyar (0.3759) than in Kani (0.2915).

**Table 1:** Allele frequencies and hetrozygosities at 7 *Alu* loci in Kani and Paliyar tribes of Western Ghats of Tamil Nadu, South India.

Locus	Genotype Frequencies	Kani (n = 49)	Paliyar (n = 57)
	+/+	0.5000	0.4912
*ACE	+/-	0.5000	0.4998
	-/-	0.5000	0.5087
	**heterozygosity**	**0.5918**	**0.5614**
	+/+	0.7959	0.5701
*TPA25*	+/-	0.3247	0.4900
	-/-	0.2040	0.4298
	**heterozygosity**	**0.3265**	**0.5087**
	+/+	0.4795	0.6315
*FXIIIB*	+/-	0.4991	0.4653
	-/-	0.5204	0.3684
	**heterozygosity**	**0.2244**	**0.2105**
	+/+	0.8979	0.8421
*CD4*	+/-	0.1831	0.2644
	-/-	0.1020	0.1570
	**heterozygosity**	**0.1224**	**0.1754**
	+/+	0.7857	0.5964
*APO*	+/-	0.3367	0.4812
	-/-	0.2142	0.4035
	**heterozygosity**	**0.1428**	**0.3508**
	+/+	0.4387	0.5438
*D1*	+/-	0.4924	0.4961
	-/-	0.5612	0.4561
	**heterozygosity**	**0.1020**	**0.3859**
	+/+	0.5306	0.6052
*PV92*	+/-	0.4980	0.4778
	-/-	0.4693	0.3947
	**heterozygosity**	**0.5306**	**0.4385**
All loci	**heterozygosity**	**0.2915**	**0.3759**

*ACE: Angiotensin Converting Enzyme; *TPA25*: Tissue Plasminogen activator 25; *FXIIIB*: Improved Coagulation Factor XIIIB; *CD4*: Cluster of Differentiation 4; *APO*: Apolipoprotein; *D1*:Alu insertion D1; *PV92*:*Alu* insertion *PV92*

### Genomic Diversity between Tribal Populations

The genetic differentiation or genetic differences among tribal populations, Gst values (a measure of the interpopulation variability) for each *Alu* locus was determined. The gene diversity analysis was performed based on the polymorphism data for eleven tribal populations, two from the present study (Kani and Paliyar), five South Indian Tribes (SITs) such as Badga, Irula, Kota, Kurumba and Toda [24] and four North Indian Tribes (NITs) such as Lodha, Munda, Santal and Tipperah [25] from previous studies (Table 2). The total genomic diversity (Ht) in all the populations studied was high except for CD4 locus. The Gst value ranges from 5.7 (minimum) for CD4 to 10.4 (maximum) for APO in SITs and from 3.9 for D1 to 16.8 for APO in NITs. Thus the ‘range of genetic variability’ is broader for NITs than the SITs when the two groups were considered separately. However, for combined South and North Indian tribes (CSNITs), Gst value ranges from 6.0 for CD4 to 16.5 for APO. When all the loci are jointly considered, the total genetic diversity is 8.3% for SITs, 7.3% for NITs and 9.5% for CSNITs (Table 2).

**Table 2:** Gene diversity analysis of individual loci, and all loci considered jointly in tribal populations.

Locus	Ht			Hs			Gst		
	SIT*	NIT*	CSNIT*	SIT	NIT	CSNIT	SIT	NIT	CSNIT
***ACE***	0.48539	0.47991	0.47590	0.45038	0.44763	0.4400	0.07212	0.06726	0.0753
***FxIII B***	0.45151	0.42369	0.42350	0.41058	0.39324	0.3880	0.09065	0.07186	0.0836
***CD4***	0.09986	0.09859	0.07370	0.09412	0.09197	0.0693	0.05750	0.06712	0.0600
***APO***	0.39732	0.46798	0.43630	0.35581	0.38898	0.3642	0.10445	0.16879	0.1651
***Pv92***	0.49817	0.48481	0.49940	0.45311	0.46197	0.4516	0.09046	0.04711	0.0958
***TPA 25***	-	0.47886	-	-	0.45235	-	-	0.05536	-
***D1***	-	0.44524	-	-	0.463420	-	-	0.03921	-
**All loci**	0.38645	0.41129	0.38180	0.35284	0.38565	0.3426	0.08304	0.07381	0.0959

*ACE*: Angiotensin Converting Enzyme; *TPA25*: Tissue Plasminogen activator 25; *FXIIIB*: Improved Coagulation Factor XIIIB; *CD4*:Cluster of Differentiation 4; *APO*:Apolipoprotein; *D1*:Alu insertion D1; *PV92*:*Alu* insertion *PV92*: *H*t–Total genomic diversity among the populations; *H*s–Diversity between individuals within population; *G*st–Genetic diversity between population;

* **SIT–**South Indian Tribe; **NIT**-North Indian Tribe; **CSNIT**-Combined South and North Indian tribe

### Allele frequencies in Caste Populations

The allele frequencies for the insertion (+) and deletion (-) for loci *Alu* ACE, *TPA25*, FXIIIB, *APO*, *D1*, Pv92 and CD4 of 12 caste populations were presented in Table 3. *Alu* CD4 exhibits low level of polymorphism in many of the populations studied. All the populations showed very high levels of polymorphism for all *Alu* loci except CD4. Among 12 population groups studied, the heterozygosity of seven loci ranged between 0.291 (Kani)—0.468 (Yadava).

**Table 3:** Average hetrozygosities based on 7 *Alu* loci in caste/tribal populations from South India.

Name of the population	*ACE*	*Tpa 25*	*FxIII B*	*CD4*	*APO*	*D1*	*Pv92*	All Loci
Pallan (n = 50)	0.4130	0.4782	0.4782	0.1521	0.3043	0.3478	0.5217	0.3850
Nair (n = 56)	0.6041	0.5535	0.4464	0.1785	0.4107	0.2678	0.5892	0.4357
Namboothiri (n = 35)	0.2894	0.4210	0.5000	0.1052	0.7631	0.4210	0.4736	0.42477
Kani (n = 49)	0.5918	0.3265	0.2244	0.1224	0.1428	0.1020	0.5306	0.29152
Vanniyar (n = 51)	0.5600	0.4600	0.4000	0.1800	0.2200	0.1200	0.4000	0.33428
Paliyar (n = 58)	0.5614	0.5087	0.2105	0.1754	0.3508	0.3859	0.4385	0.37590
Narikuravar (n = 41)	0.6097	0.1707	0.3658	0.2439	0.3658	0.2439	0.3414	0.33447
Sourastra (n = 39)	0.4102	0.4615	0.3846	0.1025	0.2307	0.3333	0.5128	0.34797
Iyer (n = 44)	0.4883	0.4186	0.2790	0.1395	0.1627	0.2352	0.5581	0.32594
V.Goundar (n = 44)	0.5227	0.5681	0.4772	0.0909	0.2045	0.5227	0.5227	0.41556
Kallar (n = 53)	0.6425	0.5476	0.4761	0.1190	0.5238	0.1904	0.2380	0.39110
Yadava (n = 54)	0.5000	0.8148	0.3703	0.0363	0.6666	0.4363	0.4545	0.46845

ACE: Angiotensin Converting Enzyme; Tpa25: Tissue Plasminogen activator 25; FxIIIB: Improved Coagulation Factor XIIIB; CD4: Cluster of Differentiation 4; APO: Apolipoprotein; D1: Alu insertion D1; PV92: Alu insertion PV92

### Genomic Diversity between Populations

Gst values for each polymorphic locus were determined among populations and the results were presented separately for each locus and also for all loci taken together (Table 4). The total genomic diversity (Ht) among the populations was quite high. The Ht value ranged between 0.255 (CD4) to 0.499 (D1). When all loci are jointly considered between populations the total genetic diversity (Gst) was 3.6%.

**Table 4:** Analysis of gene diversity for individual loci considered jointly between populations.

*Alu* Locus	Ht	Hs	Gst
*ACE*	0.4959	0.4664	0.0595
*TPA 25*	0.4784	0.4572	0.0444
*FxIII B*	0.4951	0.5219	0.0494
*CD4*	0.2550	0.1485	0.0109
*Apo*	0.4336	0.4153	0.0420
*D1*	0.4994	0.4871	0.0246
*Pv92*	0.4900	0.4773	0.02602
All Loci	0.4496	0.4248	0.03671

*H*t–Total genomic diversity among the populations.

*H*s–Diversity between individuals within population.

*G*st–Genetic diversity between population.

### Genetic Affinities among Tribal Populations

**Fig 2:**
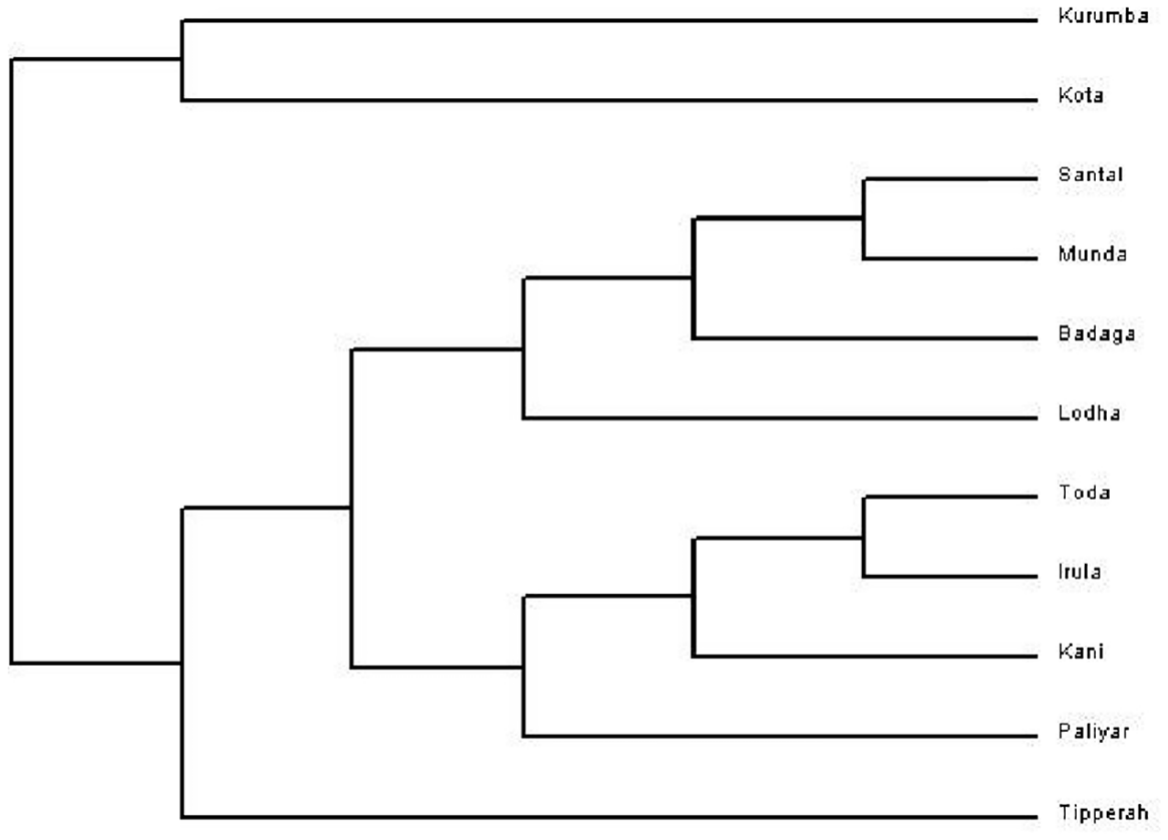
Neighbor- joining tree of tribal populations based on *Alu* polymorphism.

The genetic affinities among eleven tribal groups, 2 tribal populations of the present study namely Kani and Paliyar and the 9 tribal populations studied previously [24, 25] were reconstructed using the neighbor-joining (NJ) method (Fig 2). The maximum-likelihood tree, revealed that the Dravidian speaking South Indian tribes Kurumba and Kota exhibiting close genetic affinities. The Tibeto-Burman speaking Tipperah stand out as a unique genetic entity, while Santal and Munda of Central India showed close genomic affinities. The South Indian Toda and Irula formed a clearly distinct cluster as was evident from the dendrogram which includes another South Indian tribal group Kani. The Paliyar overlaps with Kani cluster while another South Indian tribe Badga, overlaps with a North Indian Austro-Asiatic speaking Santal and Munda tribes. These different levels of clustering of North and South Indian tribal groups is highly interesting and supports different levels of admixture due to the historical and migrational histories, of populations of India. Thus the genetic diversity was higher among tribes than the castes when all the loci are jointly considered. Thus, our findings supported the fact that the tribes are isolated from the caste groups for long in Indian subcontinent.

### Genetic Affinities among Endogamous Caste Populations

**Fig 3:**
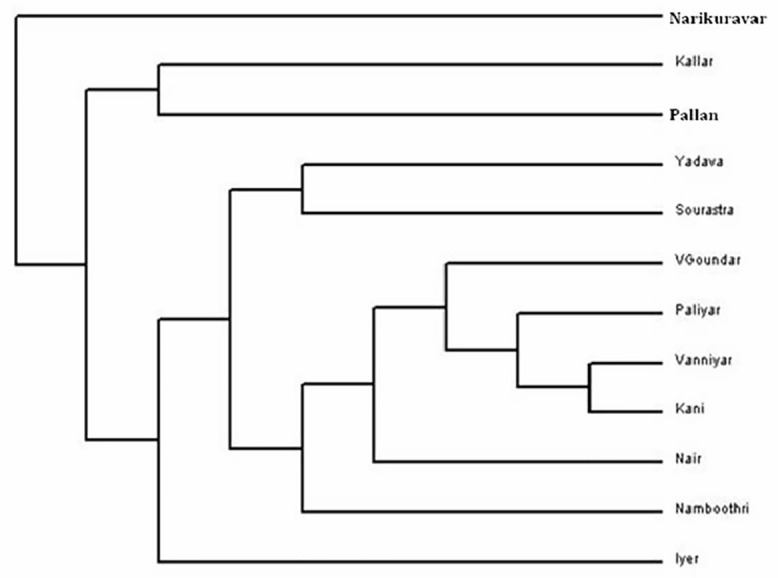
Neighbor joining tree of 10 caste and 2 tribal populations of Tamil Nadu based on *Alu* polymorphisms.

The phylogenetic relationship of 10 caste and 2 tribal population groups studied in the present work was presented in Fig 3. The 12 population groups from South India were grouped themselves in 7 clusters: (i) Kallar and Pallan cluster; (ii) Yadava and Sourashtra cluster; (iii) Vanniyar, Kani, Paliyar and Vettuva Gounder cluster; (iv) Nair cluster; (v) Namboothiri cluster; (vi) Iyer cluster; and (vii) Narikuravar cluster formed a separate cluster.

**Fig 4:**
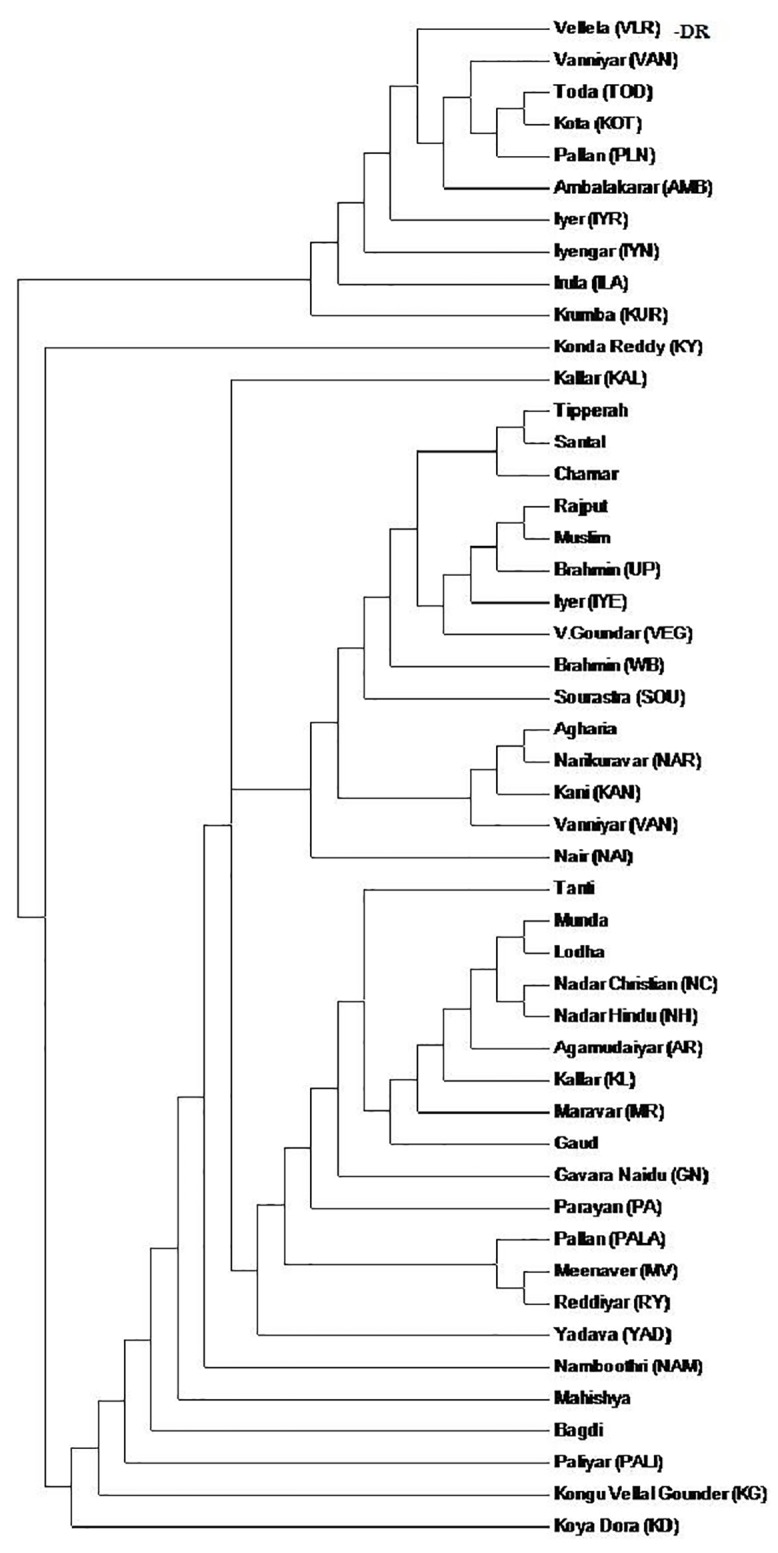
Neighbor-joining tree of 48 caste populations of India based on allele frequency data of 7 loci.

**Fig 5:**
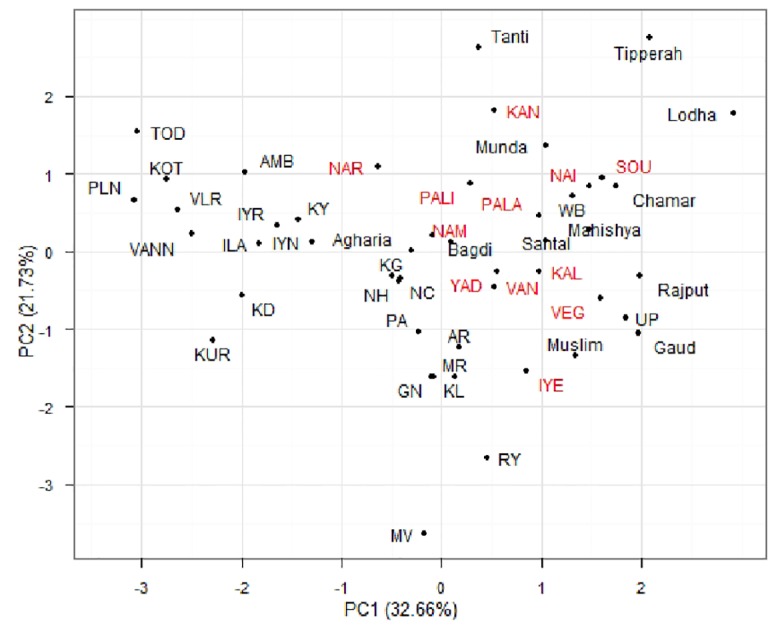
Principal component analysis of 48 caste populations of India based on allele frequency data (Red color indicates present study populations).

The genetic relationships of these ethnic populations of Tamil Nadu castes and tribes were compared with other Indian populations using the polymorphic data on seven *Alu* insertion marker [21, 23, 32]. The NJ tree of 48 populations (including 12 populations from the present study and 36 populations from the previous studies (S2 Table) was presented in Fig 4. A PCA plot for 48 caste and tribal Indian populations was constructed. The total variance analysis of allele frequencies were 33.26 and 21.66% respectively for PC1 and PC2 for the seven polymorphic *Alu* insertion loci (Fig 5).

## Discussion

Of late, the *Alu* polymorphisms studies have gained importance in the making of genetic structure of human populations because of the fact as new alleles are not generated and as there is no selection pressure acting upon these loci. The present work was carried out to explore the genetic variations at a number of polymorphic *Alu* loci among tribal and caste populations from the state of Tamil Nadu, South India (77° and 80° E latitude and 8° and 13° N longitude). The allele frequencies and heterozygosity v*Alu*es observed in the present study are comparable, however with minor deviations with the frequencies of other South Indian endogamous populations studied previously. The overall average heterozygosity of all the loci analyzed ranges between 0.292–0.468. The lowest level of heterozygosity in the present study was observed in Kani (0.292), a primitive tribe inhabiting Western Ghats (altitude: 2,695, mts above the sea level) Tamil Nadu. A classical study was undertaken by Watkins et al [33] to elucidate the extent of genetic differentiation of Indian populations and to trace their ancestry. This study place South Indian caste and tribal populations between European and East Asian populations. Further, this study also documented a relatively high between-group differentiation among Indian tribal groups, probably attributable to geographic and reproductive isolation (‘taboo’ or ‘totem’) and subsequent drift [34]. Previously, the Kani tribes have documented with less number of HLA alleles (immune response genes) [35]. High level of homozygosity in a number of HLA loci could be the reason for the reduced polymorphism. Thus, the observed low level of heterozygosities at *Alu* and HLA loci were attributed to isolation from other populations for considerably long periods of time and entertaining a tribal life in hill regions. The heterozygosity v*Alu*e of Kani (0.292) was very close to Vysyas (0.299) reported previously by Watkins et al., [33]. Nonetheless, the caste populations showed a high level of heterozygosity that ranges between 0.325–0.469. The average heterozygosity of South Indian caste populations was similar to the v*Alu*es (0.351–0.449) observed for different population groups of India [20, 24–26, 33].

The observed average genetic differences (Gst) within the each caste populations of the present study was 3.6%. Stoneking et al. [20] have reported a Gst value of 8.8% for Africans, 5.8% for South East Indians, 3.6% for Western Asians, 1.1% for Europeans and 0.1% for Australians and New Guniea populations [20]. In the present study, the average Gst value (3.6%) was much higher than the Europeans, and Australians and lower than the Southeast Indians and Africans and rather surprisingly, it matches exactly with Western Asians (3.6%). Similar Gst value (3.4%) was documented for Tamil Nadu caste populations studied earlier for *Alu* loci such as *mtNUC*, *Alu ACE*, *Alu APO*, *Alu FXIIIB*, *Alu D1*, *Alu CD4*, *Alu PLAT*, *Alu TPA25*, *Alu PV92* [32] and Western Indian populations (3.6%) studied previously [20]. Thus, our study strongly confirmed the genetic similarities between West Asian and Tamil Nadu populations studied earlier and supported the large scale coastal migrations of African populations into India through West Asian corridor. In a study, Watkins et al. [33] have reported 2.4% Gst for 12 Indian populations [33]. Previous report on castes and tribes of Andhra Pradesh and South India, have reported the average Gst value 4.8% [27]. However, the Gst of North Indian populations supposedly originated from Indo-Europeans, was observed to be 6.8% [25]. Further, Vishwanathan et al. [24] have documented a Gst value of 6.7% for South Indian tribal populations [24]. However, in our analysis, the Gst value of 7 South Indian tribals (SITs) was 8.3%. Further, the average Gst among CSNITs such as North Indian Lodha, Munda, Santal (West Bengal) and Tipperah (Tripura) [25] and the South Indian Badga, Kota, Kurumba, Irula and Toda [24] and Kani and Paliyar (present study) based on different *Alu* markers was 9.5%. Thus, the present and previous studies have confirmed the higher Gst values for north and South Indian tribes confirmed the presence of different genetic elements in Indian caste and tribal populations.

Phylogenetic analysis as depicted in Fig 2 revealed that the South Indian tribe inhabiting Nilgiri hills (Western Ghats), the Badaga overlap genetically with North Indian Austro-Asiatic speaking groups Santal and Munda. This is highly interesting and striking. Previously published data sets have pointed out that the Badagas might have migrated from Central or Eastern Europe. The Y-chromosome DNA marker (NRY) based study reported that Badaga tribe have a broader *R1a* and *R1a1* Haplogroup. The *R1a1* Haplogroup have spread in people from regions of Central Europe, East Europe, Scandinavia and Punjab. These and other findings have reiterated the fact that the Badaga tribe of Nilgiri hills of South India might have originated from the Eurasia. The tribal groups presently studied such as Kani and Paliyar are linguistically similar but are living in distant geographical locations in Western Ghats, of South India. The phylogenetic analyses have revealed that these two tribes overlap genetically with two other South Indian tribes, Toda and Irula. In the present study, Paliyar tribe forms a separate cluster, and overlap with North Indian Bagdi which belongs to Indo-Aryan ancestry. Kani tribe also overlaps with North Indian middle class Agharia and South Indian Narikuravars (an economically low and nomadic group), two populations that possess Indo-Aryan ancestry. The Narikuravars speak an Indo-Aryan language called *Vagriboli* which is a western Indian language (regions of Gujarat, Rajasthan and Maharashtra) belongs to the Indo-Aryan linguistic family. *Alu* marker based studied have documented that the North Indians are genetically highly diverse populations with variations scattered between individuals. With a glut of human migratory episodes and admixtures, the paternal genealogy of North Indians have revealed the genetic foot-prints and legacy of the Indo-Aryan speaking populations.

The Principal Components Analysis provides alternative methods for examining the inter-population relationships. The present study of endogamous caste populations revealed close relationships and/or proximity in PCA as well as in NJ phylogenetic tree. The results suggested that these populations might have a common ancestry. It is highly interesting to note that an upper class population, Iyers of Madurai, overlaps genetically with UP-Brahmin. Balakrishnan et al. [36] have reported that, Iyers of Madurai, anthropologically a western Brachycephal Armenoids, having HLA similarities with many of the south East Asians, originated either from the Eurasian steppes or Central Asia [36]. Interestingly, the middle class Vettuva Gounder overlaps genetically with Iyers. Further, the Sourashtrans, an Indo-European language speaking population group that migrated from Gujarat region of West India to Tamil Nadu overlaps genetically with upper class Brahmins of West Bengal. The upper class Brahmins of Kerala, Namboothiris show a separate cluster as outer elements. Nairs overlap and cluster with North Indian endogamous groups and not with Namboothiries of Kerala. Previous studies on Nairs have reported that, they are more similar to Western European populations. Interestingly, the Piramalai Kallar population from Madurai (Tamil Nadu state, South India) forms a separate cluster as an outer branch of NJ tree. One previous study [37] have reported that, the homeland of Piramalai Kallars was somewhere in the Middle East [37]. It is possible to believe that they might have come in the first ‘Out-of-Africa’ migration to India, moved further and settled in south India (particularly in Madurai region). Anthropologically, Piramalai Kallars belongs to the Major Group-II (non-Brahmin low rank), thought to be of paleo-Mediterranean origin [38, 39]. The clustering of castes such as Vanniyar, Pallan, Iyer and Kallar at two different points in the dendrogram/phylogenetic tree could be due to differences in the strategies of sampling and/or genotyping methodologies adopted by various research groups. These issues need to be addressed in a future multicentric study.

Indian sub-continent has witnessed a massive gene flow from varied ethnic sources over the historical periods. The gene flow could be occurred prior to the subdivision of this population into largely endogamous caste groups. Thus, it was suggested that, after the migration of modern humans from Asia, there were many rapid population explosion (s) following an initial period of isolation. To conclude, the studied Indian populations have revealed higher heterozygosities as compared to African populations. The present study thus concluded that the endogamous populations of South India have showed the amalgamation of various populations coming–in from different directions and geographical locations by the process of admixture and miscegenation. Thus, the *Alu* polymorphism based affinities of the South Indian populations (castes and tribes) forms a potential genetic data for mapping population migrations, histories, genetic similarities and gene-disease linkage analysis in a country known for the practice of strict endogamy and higher level of prevalence of infectious diseases. Our study thus provides (i) an evidence of presence of North/South differences in the frequencies of *Alu* alleles and (ii) affinities of South Indian endogamous caste and tribes with middle East and West Asian populations. These observations thus confirmed the well established notion of peopling of South India by coastal migrations of man from Africa.

## Supporting Information

S1 File Ethnographic Notes on the Samples Studied. (DOCX)

S1 Table Ethnographic Notes on the Samples Studied. (DOC)

S2 Table List of previous study population were used in the present study. (DOC)

## References

[1] Cann RL (2001) Genetic clues to dispersal in human populations: retracing the past from the present. Science 291: 1742–1748. 11249820 10.1126/science.1058948

[2] Bhasin MK and Walter H (2001) Genetics of Castes and Tribes of India. Kamla Raj Enterprises, Delhi.

[3] Census of India. 2001. Available: http://censusindia.gov.in/.

[4] Cavalli-Sforza LL, Menozzi P, Piazza A (1994) The history and geography of human genes. Princeton University Press Princeton. N.J. 1994.

[5] Balakrishnan V, Sanghvi D, Morphological and genetic distances in Tamil Nadu In: Sanghvi LD, Balakrishnan V, Karve I (Eds) (1981): Biology of the People of Tamil Nadu. Bombay: The Indian Society of Human Genetics, Calcutta: The Indian Anthropological Society, Calcutta 103–143.

[6] Reich D, Thangaraj K, Patterson N, Price AL and Singh L (2009) Reconstructing Indian population history. Nature: 461: 489–494. doi: 10.1038/nature08365 19779445 10.1038/nature08365 PMC2842210

[7] Ullu E, Murphy S, Melli M (1982). Human 7S RNA consists of a 140 nucleotide middle repetitive sequence inserted in an *Alu* sequence. Cell; 29: 195–202. 6179628 10.1016/0092-8674(82)90103-9

[8] Rogers J (1983) Retroposons defined. Nature 1983; 301: 460. 6185851 10.1038/301460e0

[9] Houck CM, Rinehart FP, Schmid CW (1979) A ubiquitous family of repeated DNA sequences in the human genome. J Mol Biol: 132: 289–306. 533893 10.1016/0022-2836(79)90261-4

[10] Lander ES, Linton LM, Birren B, Nusbaum C, Zody MC, Baldwin J, et al (2001) Initial sequencing and analysis of the human genome. Nature: 409: 860–921. 11237011 10.1038/35057062

[11] Batzer MA, Kilroy GE, Richard PE, Shaikh TH, Desselle TD, Hoppens CL, et al (1990) Structure and variability of recently inserted *Alu* family members. Nucleic Acids Res: 18: 6793–6798. 2175877 10.1093/nar/18.23.6793 PMC332733

[12] Batzer MA, Deininger PL (1991) A human-specific subfamily of *Alu* sequences. Genomics: 9: 481–487. 1851725 10.1016/0888-7543(91)90414-a

[13] Economou EP, Bergen AW, Warren AC, Antonarakis SE (1990) The polydeoxyadenylate tract of *Alu* repetitive elements is polymorphic in the human genome. Proc Natl Acad Sci: 87: 2951–2954. 2326257 10.1073/pnas.87.8.2951 PMC53811

[14] Novick GE, Batzer MA, Deininger PL, Herrera RJ (1996) The mobile genetic element *Alu* in the human genome. BioScience: 46: 32–41.

[15] Rowold D, Herrera RJ (2000) *Alu* elements and the human genome. Genetica: 108: 57–72. 11145422 10.1023/a:1004099605261

[16] Ray DA, Xing J, Hedges DJ, Hall MA, Laborde ME, Anders BA, et al (2005) *Alu* insertion loci and platyrrhine primate phylogeny. Mol Phylogenet Evol: 35: 117–126. 15737586 10.1016/j.ympev.2004.10.023

[17] Xing J, Wang H, Han K, Ray DA, Huang CH, Chemnick LG, et al (2005) A mobile element based phylogeny of Old World monkeys. Mol Phylogenet Evol: 37: 872–880. 15936216 10.1016/j.ympev.2005.04.015

[18] Salem AH, Kilroy GE, Watkins WS, Jorde LB, Batzer MA (2003) Recently integrated *Alu* elements and human genomic diversity. Mol Biol Evol: 20: 1349–1361. 12777511 10.1093/molbev/msg150

[19] Batzer MA, Stoneking M, Alegria-Hartman M, Bazan H, Kass DH, Shaikh TH, et al (1994) African origin of human-specific polymorphic *Alu* insertions. Proc Natl Acad Sci USA: 91: 12288–12292. 7991620 10.1073/pnas.91.25.12288 PMC45422

[20] Stoneking M, Fontius JJ, Clifford SL, Soodyall H, Arcot SS, Saha N et al (1997) *Alu* insertion polymorphisms and human evolution: evidence for a larger population size in Africa. Genome Res: 1061–1071. 9371742 10.1101/gr.7.11.1061 PMC310683

[21] Majumder PP, Roy B, Banerjee S, Chakraborty M, Dey B, Mukherjee Roy, et al (1999b) Human-specific insertion/deletion polymorphisms in Indian populations and their possible evolutionary implications. Eur J Hum Genet: 7: 435–446. 10352934 10.1038/sj.ejhg.5200317

[22] Watkins WS, Ricker CE, Bamshad MJ, Carroll ML, Nguyen SV, Batzer MA, et al (2001) Patterns of ancestral human diversity: an analysis of *Alu*-insertion and restriction site polymorphisms. Am J Hum Genet: 68: 738–752. 11179020 10.1086/318793 PMC1274485

[23] Basu A, Mukherjee N, Roy S, Sengupta S, Banerjee S, Chakraborty M, et al (2003) Ethnic India: A genomic view, with special reference to peopling and structure. Genomic Res: 13:2277–2290. 10.1101/gr.1413403 PMC403703 14525929

[24] Vishwanathan H, Deepa E, Cordaux R, Stoneking M, Usha Rani MV, Majumder PP (2004) Genetic structure and affinities among tribal populations of southern India: a study of 24 autosomal DNA markers. Annals of Human Genetics: 68:128–138. 15008792 10.1046/j.1529-8817.2003.00083.x

[25] Majumder PP, Roy B, Banerjee S, Chakraborty M, Dey B, Mukherjee N, et al (1999) Human-Specific insertion deletion polymorphisms in Indian populations and their possible evolutionary implicatins. Eur J Hum Gen: 7: 435–446. 10.1038/sj.ejhg.5200317 10352934

[26] Veerraju P, Rao TV, Lakshmi N, Reshmi S, Badal Dey, Majumder Partha P (2001) Insertion/deletion DNA polymorphisms in two South Indian tribal populations. Int J Hum Genet: 1: 129–132.

[27] Vishwanathan H, Edwin Deepa, Usharani M. V, Majumder P (2003) Insertion/Deletion Polymorphisms in Tribal Populations of Southern India and Their Possible Evolutionary Implications. Human Biology Volume 75, Number 6, 12, p.873–887. 15018036 10.1353/hub.2004.0013

[28] Mukherjee N, Mitra M, Chakraborty M, Majumder Partha P (2000) Congruence of genomic and ethnolinguistic affinities among five tribal populations of Madhya Pradesh, India. J. Genet: 79:41–16.

[29] Welsh KI, Bunce M (1999) Molecular typing for the MHC with PCR-SSP. Rev Immunogenet: 1: 157–176. 11253945

[30] Batzer MA, Rubin CM, Hellmann-Blumberg U, Alegria-Hartman M, Leeflang EP, Stern JD, et al (1995) Dispersion and insertion polymorphism in two small subfamilies of recently amplified human *Alu* repeats. J. Mol. Biol: 247: 418–427. 7714898 10.1006/jmbi.1994.0150

[31] Nei M (1973) Analysis of gene diversity in subdivided populations. Proc Natl Acad Sci: 70: 3321–3323. 4519626 10.1073/pnas.70.12.3321 PMC427228

[32] Kanthimathi S, Vijaya M, Ramesh A (2008) Genetic study of Dravidian castes of Tamil Nadu. Journal of Genetics: 87: 2. 10.1007/s12041-008-0027-1 18776648

[33] Watkins WS, Rogers AR, Ostler CT, Wooding S, Bamshad MJ, Brassington AM, et al (2003). Genetic variation among world populations: inferences from 100 *Alu* insertion polymorphisms. Genome Res. 13(7):1607–18. 12805277 10.1101/gr.894603 PMC403734

[34] Das K, Malhotra KC, Mukherjee BN, Walter H, Majumder PP and Papiha SS (1996). Population structure and genetic differentiation among 16 tribal populations of central India. Hum. Biol. 68: 679–705. 8908797

[35] Balakrishnan K, Rathika C, Kamaraj R, Subashini R, Saravanan MP, Asha KV et al (2012). Gradients in Distribution of HLA—DRB1* Alleles in Castes and Tribes of South India. Int J Hum Genet, 12(1), 45–55.

[36] Balakrishnan K, Pitchappan RM, Suzuki K, Kumar US, Santhakumari R, Tokunaga K (1996) HLA affinities of Iyers, a Brahmin population of Tamil Nadu, South India. Hum Biol: 68(4):523–37. 8754259

[37] Shanmugalakshmi S, Balakrishnan K, Manoharan K, Pitchappan RM (2003) HLA-DRB1*, -DQB1* in Piramalai Kallars and Yadhavas, two Dravidian speaking castes of Tamil Nadu, south India. Tissue Antigens: 61: 451–464. 12823769 10.1034/j.1399-0039.2003.00061.x

[38] Malhotra KC, Balakrishnan V, Karve I. Anthropometric variation in Tamil Nadu In: Sanghvi LD, Balakrishnan V, Karve I (Eds): Biology of the People of Tamil Nadu. Bombay: The Indian Society of Human Genetics, Calcutta: The Indian Anthropological Society, Calcutta 1981: 50–74.

[39] Sanghvi LD, Balakrishnan V, Karve I (1981) Biology of the People of Tamil Nadu. Indian Society of Human Genetics, Pune, and The Indian Anthropological Society, Kolkata.

