# Correction: Polymorphic *Alu* Insertion/Deletion in Different Caste and Tribal Populations from South India

**DOI:** 10.1371/journal.pone.0162055

**Published:** 2016-08-25

**Authors:** Rathika Chinniah, Murali Vijayan, Manikandan Thirunavukkarasu, Dhivakar Mani, Kamaraj Raju, Padma Malini Ravi, Ramgopal Sivanadham, Kandeepan Chithan, Mahalakshmi Nithyanandam, Balakrishnan Karuppiah

The eighth author’s name is incomplete. The complete name is: Kandeepan Chithan.

The ninth author’s name is incomplete. The complete name is: Mahalakshmi Nithyanandam.

In the second sentence of the second paragraph of the Introduction, the word “profess” should say “proteins.” The correct sentence is: “The genetic variations and/or polymorphism at loci that code for expressed proteins are commonly deleterious and therefore, are often negatively selected and hence eliminated.

There is an error in the eighth sentence of the second paragraph of the Introduction. The number “1,100,000” should instead say “100,000.” The correct sentence is: “The human genome contains 100,000 *Alu* repeats, which represents ~11% of nuclear DNA [10].”

There is an error in the caption for S1 Table. Please see the correct S1 Table caption below.

## Supporting Information

S1 Table List of Primers and cycling conditions of *Alu* loci studied (DOC)
