# Supplementary material for: Polymorphic Alu Insertion/Deletion in Different Caste and Tribal Populations from South India
Source: PLoS One. 2016 Jun 17;11(6):e0157468. doi: 10.1371/journal.pone.0157468 (PMC4912101; doi:10.1371/journal.pone.0157468)
Supplement: S1 File — (DOCX) [file pone.0157468.s001.docx]

**S1 File: Ethnographic Notes on the Samples Studied**

***1) Pallans :*** The Pallans are an ancient community, engaged extensively in wet land farming and distributed mainly in Thanjavur, Madurai, Thirunelveli, and Ramanathapuram districts (Ramaiah 2004). The Pallars are a rural community and placed lower in the social hierarchy and grouped as Adi Dravidar (ancient Dravidians) along with other socially backward communities.

***2) Nairs:*** The Nairs are believed to be the first Dravidian invaders of Kerala, who belonged to the warrior class and later assumed the position of the governing and landowning class.

3) ***Namboothiris:*** The Namboothiris are the Brahmins who migrated to Kerala recently. Claiming priestly origin, the Namboothiris established superiority over the "Patters," the Tamil Brahmins in Kerala (Malyalam region of South india).

***4) Kani Tribes:***  Kani tribes also known as Kanikkaran, Kanikkar, Velanmars, Malaiarasan and Malavedan. They inhabit the hills of Trivandrum district and also in the Papanasam hills of Tirunelveli district and Pechiparai hills of Kanyakumari district of Tamil Nadu. In Tamil Nadu part they are conversant with the Tamil language in addition to their mother tongue Malayalam and use the Tamil script for writing. Kani tribes are following a maternal lineage of inheritance. These maternal clans are called as ‘Illam’. Buchi (1953), on the basis of a serogenetic study, a postulated a relationship with the Australian aborigines (Australoids). However, blood group genetics the blood gene distribution of the Kani puts them closer to the Caucasoids.

***5)Vanniyars:***  The Vanniyar community, with a population size of 20 million is predominant in northern parts of Tamil Nadu. The Vanniyars, known as Pallis, became soldiers and commanders during the medieval [Pallava](http://en.wikipedia.org/wiki/Pallava) rule and came to be known as ‘Padayatchis’.

***6) Paliyar Tribes:*** The Paliyars are scheduled tribes living in the south Western Ghats in south India, especially in Tamil Nadu and Kerala region. They are traditional nomadic hunter-gatherers, involved in foraging and honey collecting activities.

***7) Narikuravars****:*  There are twenty seven sub-sects in itself, including Narikuravars. Among them, the most disadvantaged section is Narikuravar who still live as gypsies now placed under Most Backward Community (MBC) list of Tamil Nadu and exist today as semi-urban-settlers. The main occupation is hunting. But as they were prohibited entry into the forests to pursue their livelihood, they were forced to take up other alternatives such as selling beaded ornaments.

***8) Sourashtrans:*** Sourashtrans are mostly cotton and silk weavers and silk thread merchants, originated and migrated from the Sourashtra region (Gujarat and parts of Maharashtra states) in northern India and later settled in Madurai few centuries ago (settled by Nayak kings of Madurai).

***9) Iyer:***  Iyers are the Brahmins of Tamil Nadu. Brahmins everywhere in India enjoy the highest social status in Hindu society and claim that they are the descendants of one of the seven rishis (saints). Brahmins from different parts of India have adopted the local languages and customs of their place of settlement (e.g., first-cousin and uncle –niece marriage in south India but not in north India), although their ideologies, profession and major language (Sanskrit) remained the same. Anthropologically the Iyers are placed in Western Brachycephal Armenoids.

10) ***Vettuva Gounders:*** The Vettuva Gounders claim the lineage of Siva Bhakta Kannapa Nayanaar. Today most Vettuva Gounders live in the districts of Salem, Namakkal, Erode, Karur, [Trichy](http://en.wikipedia.org/wiki/Tiruchirappalli) and Coimbatore originally practiced a forest based livelihood activities and later as settled agricultralistics.

***11) Kallars of Tanjore:*** Kallars are a widespread, ancient population living in southern parts of Tamil Nadu. Traditionally, they were described as semi agriculturists and semi warriors. Kallars are known to be the oldest immigrants of Neolithic period with Mediterranean racial elements (Malhotra et al*.* 1981). Kallars of Thanjavur district of Tamil Nadu form one of the geographically differentiated endogamous groups of the dominant Kallar community of Tamil Nadu, who are described as a martial community in the early Chola and Pandya periods. The Tanjore Kallars comprise an endogamous subgroup of the Kallar community exhibiting exogamy at the clan level while maintaining strict endogamy at the subgroup level.

**12) Yadavas:** The Yadavas of Tamil Nadu were originally referred as ‘Aryas’ in Tamil literature but locally called as ‘Idayans’. The people of this community worshipped Lord Krishna. They are listed in ancient Indian literature as the segments of the lineage of Yadu (*Yaduvamsha*).
