# Supplementary material for: Polymorphic Alu Insertion/Deletion in Different Caste and Tribal Populations from South India
Source: PLoS One. 2016 Jun 17;11(6):e0157468. doi: 10.1371/journal.pone.0157468 (PMC4912101; doi:10.1371/journal.pone.0157468)
Supplement: S1 Table — (DOC) [file pone.0157468.s002.doc]

| ***Alu* Locus** | **Primer sequences** | **Cycling condition** |
| --- | --- | --- |
| *D1* | F:5’- TGC TGA TGC CCA GGG TTA GTA AA – 3’  R: 5’- TTT CTG CTA TGC TCT TCC CTC TC - 3’ | 30 cycles x (94^o^C for 1min, 66^o^C for 2min, 72^o^C for 2min) |
| *ACE* | F: 5’- CTG GAG ACC ACT CCC ATC CTT TCT – 3’  R: 5’- GAT GTG GCC ATC ACA TTC GTC AGA T – 3’ | 30 cycles x (94^o^C for 1min, 58^o^C for 2min, 72^o^C for 2min) |
| *APO* | F: 5’- AAG TGC TGT AGG CCA TTT AGA TTA G –3’  R:5’- AGT CTT CGA TGA CAG CGT ATA CAG A – 3’ | 30 cycles x (94^o^C for 1min, 50^o^C for 2min, 72^o^C for 2min) |
| *PV 92* | F: 5’- AAC TGG GAA AAT TTG AAG AGA AAG T – 3’  R:5’- TGA GTT CTC AAC TCC TGT GTG TTA G – 3’ | 30 cycles x (94^o^C for 1min, 54^o^C for 2min, 72^o^C for 2min) |
| *TPA 25* | F: 5’- GTA AGA GTT CCG TAA CAG GAC AGC T – 3’  R:5’- CCC CAC CCT AGG AGA ACT TCT CTT T – 3 | 30 cycles x (94^o^C for 1min, 58^o^C for 2min, 72^o^C for 2min) |
| *FX III B* | F:5’- TCA ACT CCA TGA GAT TTT CAG AAG T – 3’  R:5’- CTG GAA AAA ATG TAT TCA GGT GAG T – 3’ | 30 cycles x (94^o^C for 1min, 56^o^C for 2min, 72^o^C for 2min) |
| *CD4* | F: 5’- AGG CCT TGT AGG GTT GGT CTG ATA – 3’ 5’  R: 5’-TGC AGC TGC TGA GTG AAA GAA CTG – 3’ | 30 cycles x (94^o^C for 1min, 58^o^C for 2min, 72^o^C for 2min) |

**S1 Table. Primers and cycling conditions of the seven *Alu* loci studied**
