# Supplementary material for: Polymorphic Alu Insertion/Deletion in Different Caste and Tribal Populations from South India
Source: PLoS One. 2016 Jun 17;11(6):e0157468. doi: 10.1371/journal.pone.0157468 (PMC4912101; doi:10.1371/journal.pone.0157468)
Supplement: S2 Table — (DOC) [file pone.0157468.s003.doc]

S2 Table. List of previous study population were used in the present study

| **Caste** | **Location** | **N** |
| --- | --- | --- |
| Kongu Vellala Gounder (KG)* | Karur and Erode (Tamil Nadu)^#^ | 50 |
| Kallar (KL) | Thanjavur and Madurai (Tamil Nadu) | 54 |
| Maravar (MR) | Kamudi and Chennai (Tamil Nadu) | 68 |
| Parayan (PA) | Chennai and Cuddalore (Tamil Nadu) | 50 |
| Reddiyar RY) | Trichy (Tamil Nadu) | 49 |
| GavaraNaidu (GN) | Madurai and Virudunagar (Tamil Nadu) | 33 |
| Agamudaiyar (AR) | Mannargudi (Tamil Nadu) | 47 |
| Meenaver (MV) | Chennai (Tamil Nadu) | 50 |
| Nadar Hindu (NH) | Tuticorin and Tirunelveli (Tamil Nadu) | 50 |
| NadarChristian (NC) | Kanniyakumari and Tirunelveli (Tamil Nadu) | 46 |
| Agharia (AGG) | Sundergarh (Orissa) | 46 |
| Bagdi (BAG) | Hooghly (West Bengal)  Garhwal (Uttar Pradesh) | 62 |
| Brahmin-UP (UBR) | West Bengal | 54 |
| Brahmin-WB (WBR) | Garhwal (Uttar Pradesh) | 46 |
| Chamer (CHA) | Sundergarh (Orissa) | 36 |
| Gaud (GAU) | Hooghly (West Bengal) | 28 |
| Mahishya (MAH) | Garhwal (Uttar Pradesh) | 68 |
| Muslim (MUS) | Garhwal (Uttar Pradesh) | 50 |
| Rajput (RAJ) | Sundergarh (Orissa) | 98 |
| Tanti (TAN) | Sundergarh (Orissa) | 28 |
| Pallan (PLN) | Tiruchirapalli (Tamil Nadu) | 50 |
| Ambalakarar (AMB) | Dindigul (Tamil Nadu) | 50 |
| Vanniyar (VAN) | Cuddalore (Tamil Nadu) | 50 |
| Veerakodi Vellallar (VVL) | Thanjavur (Tamil Nadu) | 50 |
| Iyer (IYR) | Tiruchirapalli and Thanjavur (Tamil Nadu) | 50 |
| Iyengar (IYN) | Tiruchirapalli and Thanjavur (Tamil Nadu) | 50 |
| Irula (ILA) | Nilgiri (Tamil Nadu) | 50 |
| Kota (KOT) | Nilgiri (Tamil Nadu) | 45 |
| Kurumba (KUR) | Nilgiri (Tamil Nadu) | 54 |
| Toda (TOD) | Nilgiri (Tamil Nadu) | 50 |
| Badaga (BAD) | Nilgiri (Tamil Nadu) | 51 |
| Koya Dora | Andra Pradesh | 55 |
| Konda Reddi | Andra Pradesh | 56 |

Cf: Kanthimathi et al.and2008and Majumder et al.and 1999; Veerraju et al.and 2001; Basu et al.and 2003.

N: number of Individuals studied. * Caste code; # State name
